# Prognostic value of liver stiffness measurement for complications after allogeneic transplant with post-transplant cyclophosphamide

**DOI:** 10.3389/fimmu.2025.1699219

**Published:** 2026-01-23

**Authors:** Rebeca Bailén, Manuel Fernández-Villalobos, Sonia Alonso, Ignacio Gómez-Centurión, Paula Fernández-Caldas, Lucía Castilla, María José Llácer, Adriana Ahumada, Carlos Iniesta, Diego Rincón, Cristina Muñoz, Santiago Sabell, Diego Carbonell, Javier Anguita, Ramón García-Sanz, Rafael Bañares, Mi Kwon

**Affiliations:** 1 Department of Hematology and Hemotherapy, Hospital General Universitario Gregorio Marañón, Madrid, Spain; 2 Instituto de Investigación Sanitaria Gregorio Marañón (IiSGM), Madrid, Spain; 3 Department of Gastroenterology, Hospital General Universitario Gregorio Marañón, Madrid, Spain; 4 Department of Medicine, Universidad Complutense de Madrid, Madrid, Spain

**Keywords:** FibroScan, graft-versus host disease, liver stiffness measurement, post-transplant cyclophosphamide, veno-occlusive disease

## Abstract

**Introduction:**

Pre-existing comorbidities prior to allogeneic hematopoietic stem cell transplantation (HSCT), significantly affects outcomes. Prior hepatic impairment is included in classic prognostic scores like HCT-CI, without taking into consideration modern assessment techniques such as liver stiffness (LS) measurement. We aimed to evaluate the value of LS using Fibroscan (FS) to predict transplant outcomes and hepatic complications in patients undergoing allo-HSCT using post-transplant cyclophosphamide (PTCY) for graft-versus-host disease (GVHD) prophylaxis.

**Methods:**

We conducted a single-center, prospective, observational study to evaluate the utility of LS measurement performed prior to transplantation and on day +14 to predict transplant outcomes, between October 2021 and March 2024. ROC curves were used to identify cut-off points for LS values for the development of hepatotoxicity, veno-occlusive disease (VOD), and hepatic acute and chronic GVHD. Logistic regression was used to analyse the impact of LS on overall survival (OS), event-free survival (EFS), non-relapse mortality (NRM) and graft-versus-host-disease and relapse-free survival (GRFS) .

**Results:**

One hundred eight patients were included. Median follow-up was 12.5 months. OS, EFS, GRFS, cumulative incidence of relapse and NRM at 12-months were 75%, 68%, 55%, 22% and 9%, respectively. Cumulative incidences of grade II-IV acute GVHD at day 180 and moderate-severe chronic GVHD at 12 months were 14% and 12%, respectively. Five patients (4.6%) developed VOD. LS variation (FSΔ) from baseline LS to day +14 was significantly increased in those patients who developed VOD compared to those who did not (p=0.048; AUROC 0.8). Logistic regression univariate analysis showed FS+14>6KPa to be predictive for worse OS and EFS (p<0.05). Multivariate analysis found FS+14>6 KPa to be predictive for worse EFS.

**Discussion:**

In our experience, increase in LS between baseline and day +14 was predictive for VOD. In addition, a measurement of FS+14>6Kpa was predictive for the outcome of allo-HSCT, with an independently predictive value for worse EFS. Thus, FS+14>6 KPa should be considered in future prognostic models used for PTCY-based HSCT.

## Introduction

Allogeneic hematopoietic stem cell transplantation (allo-HSCT) remains the only curative treatment for a wide range of hematological disorders (1). However, post-transplant complications, particularly hepatic disturbances, are common and can significantly impact outcomes. These include drug-induced liver injury (DILI) associated to conditioning regimens and other drugs, sinusoidal obstruction syndrome/veno-occlusive disease (VOD) and liver involvement of both acute and chronic graft-versus-host disease (GVHD), among others.

Liver stiffness (LS), assessed by transient elastography (TE), is a non-invasive, reproducible, and widely available tool for estimating liver fibrosis and portal hypertension in patients with chronic liver disease (2, 3). In the context of allo-HSCT, LS has been explored for early detection and monitoring of VOD (4–6) as well as for other hepatic complications (7–10). Moreover, baseline LS has been proposed as a potential tool for tailoring of conditioning regimens (11). Despite its utility, LS is not currently included in widely used comorbidity scores such as the Hematopoietic Cell Transplantation–Comorbidity Index (HCT-CI) (12).

Most studies evaluating the prognostic value of LS have focused on patients undergoing HLA-matched transplants with conventional GVHD prophylaxis typically consisting of methotrexate and a calcineurin inhibitor. However, GVHD prevention is evolving and post-transplant cyclophosphamide (PTCY) is increasingly being used not only in haploidentical settings but also in matched donor transplants (13–15). Given that PTCY introduces an additional alkylating agent, it may modify the risk of hepatic complications, potentially influencing the predictive performance of currently available comorbidity scores. Recently, the European Bone Marrow Transplant (EBMT) group has validated the impact of individual dysfunction key organs in the setting of PTCY demonstrating that the inclusion of comorbidities such as cardiac, renal, severe hepatic, severe pulmonary and infection significantly increased the risk of non-relapse mortality (NRM) whereas other comorbidities included in the HCT-CI score did not show a similar effect (16). However, hepatic dysfunction in this study was defined using conventional biochemical markers (bilirubin, transaminases) or known chronic liver disease, without incorporating LS measurements.

The aim of this study was to determine whether LS measurements obtained by TE at baseline and on day +14 can predict post-transplant complications, including hepatic events and non-relapse mortality, in patients undergoing allo-HSCT with PTCY-based GVHD prophylaxis.

## Patients and methods

### Study design

This was a prospective, single-center study conducted between October 2021 and March 2024 at a tertiary referral center specialized in complex hematologic and liver disorders.

### Patients and study procedures

The study included consecutive adult patients who underwent allo-HSCT with PTCy as GVHD prophylaxis. Predefined exclusion criteria included obesity (body mass index [BMI] > 40) and baseline ascites, which precluded reliable TE measurements.

#### Transplant procedures

Most frequently used myeloablative conditioning (MAC) consisted of Fludarabine (Flu) 40 mg/m2/day on days -6 to -3 and IV Busulfan (Bu) 3.2 mg/kg/day days -6 to -3 or -6 to -4 (FluBu3/FluBu4). Total body irradiation (TBI)-based conditioning with fludarabine in patients with acute lymphoblastic leukemia (ALL). Reduced intensity conditioning (RIC) regimens were used in patients who were either older than 60 years, showed an HCT–CI ≥ 3 or had a prior HSCT. RIC schemes consisted on a modified Baltimore protocol with Flu 30 mg/m2/day on days -6 to -2, Cy 14.5 mg/m2/day on days -6 and -5 and IV Bu 3.2 mg/kg/day days -3 to -2 (FluBuCy) for patients receiving an haploidentical transplant, and either FluBu2 or FluMel with melphalan 140 mg/m2 on day -2, adjusted to 100 mg/m2 in patients with creatinine clearance less than 60 mL/min in HLA-matched transplants. Use of treosulfan was limited to patients with significant comorbidities.

GVHD prophylaxis included IV cyclophosphamide (Cy) 50 mg/kg at days +3 and +4 combined with IV tacrolimus (FK) 0.02 mg/kg/day from day +5 and mycophenolate mofetil 10mg/kg every 8 hours from day +5 until day +35. Patients undergoing haploidentical or mismatched unrelated donor (MMUD) with low relapse risk and high risk of complications (age-adjusted HCT-CI≥3 or >55 years old) also received extended GVHD prophylaxis with abatacept 10/mg/kg IV on days +5, +14, +28 and +56. G-CSF 5 mcg/kg/day was used from day +5 until absolute neutrophil count (ANC) of 1.0×10^9^/L or greater. Prophylaxis with ursodeoxycholic acid (300mg PO/8h) was given to all patients receiving MAC regimens and all patients developing any hepatic complication.

#### Liver stiffness measurements

LS was measured by using transient elastography with the FibroScan^®^ (Echosens SA, Paris, France) device prior to conditioning and on day +14 (± 2 days). Additional measurements were performed at VOD diagnosis and during follow-up as clinically indicated. Valid measurements required ≥10 valid readings and an interquartile range-to-median ratio (IQR/M) ≤30%. Disease and transplant-related data were extracted from electronic medical records.

#### Pre- and post-transplant evaluation

Patients were stratified according to the disease risk index (DRI) (17). Pre-transplant comorbidities were evaluated using age-adjusted HCT-CI (12). Acute GVHD (aGVHD) was scored according to MAGIC criteria (18), and chronic GVHD (cGVHD) was scored according to the NIH Consensus criteria (19). Diagnosis of VOD was made according to the 2016 EBMT criteria (20). Hepatic dysfunction was graduated according to CTCAE criteria v5 (21). Liver-related events included VOD, hepatic acute and chronic GVHD or other liver dysfunctions defined by transaminitis >3 ULN or hyperbilirubinemia >2mg/dL.

Myeloid engraftment was defined as an ANC of 0.5×10^9^/L or greater for three consecutive days. Platelet engraftment was defined as a platelet count of 20×10^9^/L or higher without transfusion support for three consecutive days. Patients surviving beyond day + 28 without engraftment were evaluated for potential graft failure (GF). Disease recurrence was diagnosed based on clinical and pathological criteria.

#### Study variables

Primary endpoints included hepatic complications (VOD, hepatic acute and chronic GVHD, hepatotoxicity) and NRM. Secondary endpoints included overall survival (OS), event-free survival (EFS), myeloid and platelet engraftment, incidence of aGVHD and cGVHD, relapse and GVHD/relapse-free survival (GRFS). Relapse, toxic death and second transplantation due to GF were considered events for EFS.

### Statistical analysis

Categorical variables were expressed as frequency and percentage; continuous variables were expressed as median and either interquartile range (IQR) or range. Associations between categorical variables were assessed using the χ² test, and the non-parametric Kruskal-Wallis test was used for quantitative variables.

To evaluate the diagnostic performance of LS for the development of VOD, ROC curves were used. The best cut-off values for LS were selected using the Youden J-Index and Harrell’s C-index was used to determine the optimal LS cut-off for predictive discrimination.

Survival estimates (OS, EFS, GRFS) were calculated using the Kaplan–Meier method. Univariate and multivariate analyses were performed for the prediction of OS, EFS, NRM, relapse, and GRFS. Variables significantly associated with endpoints in univariate analysis were entered into a forward stepwise multivariate Cox regression model (p-in <0.05, p-out <0.1). Cumulative incidences were calculated in the presence of competing risks; toxic death before day +28 was considered a competing event for engraftment and NRM and relapse were treated as competing events for each other and for second transplantation. Variables included in the models are detailed in **Supplementary Table 1**. All components of the age-adjusted HCT-CI were analyzed individually in the models.

Statistical analyses were performed using SPSS (IBM SPSS Statistics for Windows, Version 21.0), Stata 17 and RStudio version 1.0.2.

### Ethics statement

The study was conducted according to the Declaration of Helsinki and approved by the Ethics Committee of the Hospital General Universitario Gregorio Marañón. Written informed consent was obtained from all participants.

## Results

### Patient and transplant characteristics

A total of 108 patients were transplanted between October 2021 and March 2024 and included in the study. Patient and transplant characteristics are summarized in **Table 1**. Median age was 58 years (range 20-72). Most frequent diagnosis were acute myeloid leukemia/myelodysplastic syndrome (52%) and acute lymphoblastic leukemia (22%). Donor was haploidentical in 46%, matched related in 22%, and matched unrelated 27%. Thirty-two patients (30%) received myeloablative conditioning: 29 were based on busulfan and 3 patients received TBI. 105 (97%) received peripheral blood as graft source. All patients received PTCy+CNI+MMF for GHVD prophylaxis, nineteen (18%) also received abatacept.

**Table 1:** Patient and transplant characteristics.

Baseline characteristics	Patients (n=108)
Sex (male, %)	58 (54)
Age (median, range)	58 (20-72)
Diagnosis (*n*, %):	
• AML/MDS	56 (52)
• ALL	24 (22)
• Non-Hodgkin lymphoma	8 (7)
• Hodgkin lymphoma	5 (4)
• cMPN or cMPN/MDS	6 (5)
• Aplastic anemia	2 (2)
• Others	7 (6)
Disease risk index (*n*, %)	
• Low	3 (3)
• Intermediate	25 (23)
• High/Very High	80 (74)
Age-adjusted HCT-CI score (*n*, %)	
• 0-2	41 (38)
• 3-4	36 (33)
• ≥5	31 (29)
Prior HSCT (n, %)	13 (12)
• Autologous	5 (5)
• Allogeneic	8 (7)
Prior exposure to inotuzumab ozogamicin (n, %)	2 (2)
Prior liver disease (n, %)	
• No history of liver diseases	85 (79)
• Non-alcoholic fatty liver disease	12 (11)
• HBV seropositivity	4 (4)
• HCV seropositivity	7 (6)
Donor (*n*, %)	
• Haploidentical	50 (47)
• Matched related donor	24 (22)
• Matched unrelated donor	29 (27)
• Mismatched unrelated donor	5 (5)
CNI+MMF+PTCy GVHD Prophylaxis (n, %)	108 (100)
• Abatacept extended prophylaxis	19 (18)
Stem cell source (*n*, %)	
• PB	105 (97)
• BM	3 (3)
Conditioning regimen intensity (n, %)	
• Myeloablative	32 (30)
o Fludarabine-busulfan	29 (27)
o Fludarabine-TBI	3 (3)
• Reduced intensity	76 (70)
o Fludarabine-busulfan	30 (28)
o Fludarabine-busulfan-cyclophosphamide	30 (28)
o Fludarabine melphalan	7 (6)
o Clofarabine-melphalan	6 (5)
o Fludarabine threosulfan	3 (2)

Percentages may not add up to 100 due to rounding.

ALL, acute lymphoid leukemia; AML, acute myeloid leukemia; BM, bone marrow; CNI, calcineurin inhibitor; c MPN, chronic myeloproliferative neoplasm; c MPN/MDS, chronic myeloproliferative neoplasm/myelodyspastic syndrome; HSCT, hematopoietic stem cell transplant; HCT-CI, Hematopoietic Cell Transplantation-Comorbidity Index; IQR, interquartile range; MDS, myelodysplastic syndrome; MFM, mycophenolic acid; PB, peripheral blood; PTCy; post-transplant cyclophosphamide; TBI, total body irradiation.

Thirteen (12%) patients had undergone prior HSCT, 5 (5%) allogenic and 8 (7%) autologous.

Age-adjusted HCT-CI was higher or equal to 3 in 67 patients (62%). Twelve (11%) patients had prior liver disease, four (4%) fatty liver disease, seven (6%) chronic HBV and one (1%) HCV, none of them had active replication.

### Engraftment and main transplant outcomes

**Figure 1:**
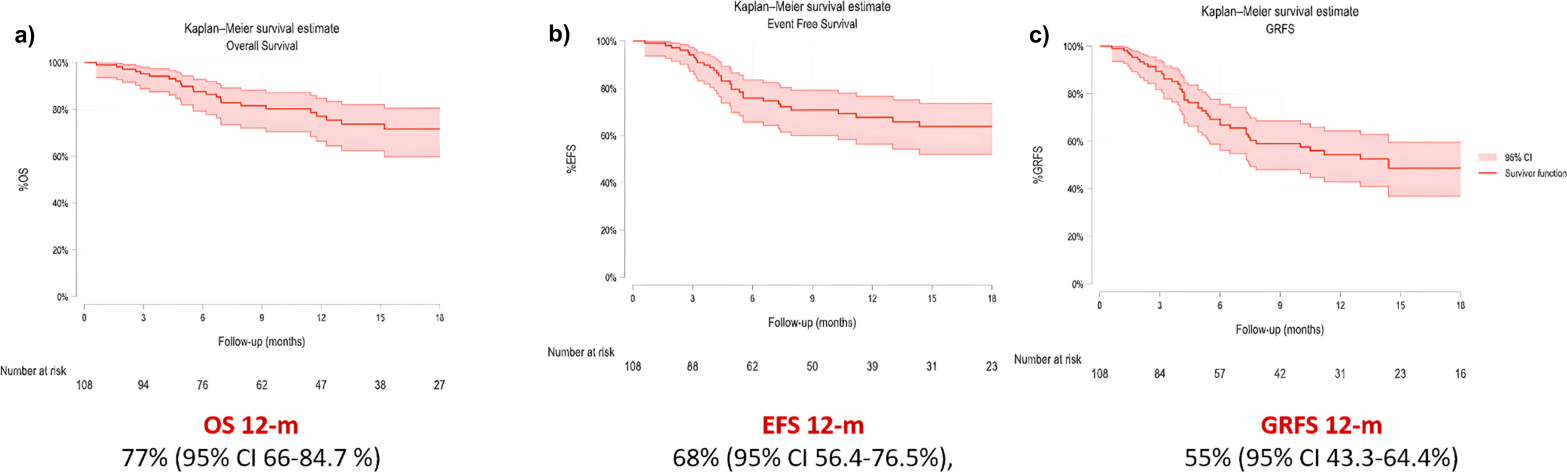
Kaplan-Meier curves for overall survival (OS) **(a)**, event-free survival (EFS) **(b)** and graft-versus-host-disease-and-relapse-free survival (GRFS) **(c)**.

The cumulative incidence of neutrophil recovery at day 28 was 94.4%; median time to myeloid engraftment was 16 days (IQR 15-19.2). The cumulative incidence of platelet engraftment at days +28 and +100 was 61% and 90% respectively. Median time to platelet engraftment was 23 days (IQR 16.5-32.5). Primary graft failure occurred in 2 patients (1.8%). With a median follow-up of 12.5 months (IQR 5.1-18.2), 12-month OS and EFS were 77% (95% CI 66-84.7%) and 68% (95% CI 56.4-76.5%), respectively. The 12-month GRFS was 55% (95% CI 43.3-64.4%) (**Figure 1**). The 12-month cumulative incidence of relapse and NRM were 22% and 9%, respectively. The cumulative incidence of acute GVHD II-IV at day +180 was 14%, and moderate-to-severe chronic GVHD at 12 months was 12%.

### Liver complications after allo-HSCT

Five patients (4.6%) developed VOD. Among them, 3 were classified as very severe, 1 as moderate and 1 as mild (**Table 2**). The mean time from SCT to VOD diagnosis was 27 days (range 15-41). No differences were found on baseline liver biochemistry in patients with and without VOD, including baseline ferritin levels (451.6 ng/mL vs 2340 ng/mL, respectively, p=0.5). Defibrotide was administrated to four patients and three patients received transjugular intrahepatic portosystemic shunt (TIPS) for VOD treatment. Four patients achieved complete VOD remission at a median of 15.5 days (IQR 12.25-16.25). Three patients died due to causes unrelated to VOD: one due to relapse (9.8 months post-VOD), and two from infections (at 44 days and 28 days post-VOD, respectively).

**Table 2:** Characteristics of patients with VOD.

Patient n°	Disease	Age, Sex	Conditioning intensity	Baseline FS (Kpa)	FS day+14 (Kpa)	FSΔ (Kpa)	FS at diagnosis (Kpa)	FS after VOD (Kpa)	Clinical VOD diagnosis(days)	EBMT 2016 severity grade	Management
1	MCL	62, M	RIC (Flu-Bu-Cy)	4.9	NA	**NA**	16		+41	Very severe	DF, TIPS
2	B-ALL	23, M	MAC (Flu-Bu)	3.9	4.6	0.7	41.1		+27	Very severe	DF, TIPS
3	B-ALL	52, F	RIC (Flu-Treo)	6	21.5	15.5		7.6	+15	Mild	Diuretics
4	T-ALL	20, F	MAC (Flu-Bu)	5.6	6.3	0.7	14.4		+30	Very severe	DF, TIPS
5	B-ALL	64, M	RIC (Flu-Bu)	14.3	24	9.7	24.3		+19	Moderate	DF

B-ALL, B-acute lymphoblastic leukemia; Bu, busulfan; Cy: cyclophosphamide; DF, defibrotide; Flu: fludarabine; MAC, myeloablative conditioning; MCL, mantle cell lymphoma; RIC, reduced intensity conditioning; T-ALL, T-cell acute lymphoblastic leukemia; TIPS, transjugular intrahepatic portosystemic shunt; Treo: treosulfan.

Acute hepatic GVHD occurred in 5 patients (4.6%) by day +180, and chronic hepatic GVHD in 8 patients (7.4%). Only one patient in each group had bilirubin >2 mg/dL. Three of the five patients with acute hepatic GVHD required systemic treatment; one did not respond and died from a severe fungal infection. The patient who did not respond to treatment received steroids, ruxolitinib and extracorporeal photopheresis (ECP), the rest only steroids. All chronic hepatic GVHD cases responded to treatment. Four patients only reintroduced CNI, one received systemic corticosteroids combined with CNI and ruxolitinib, one only ruxolitinib and two received both ruxolitinib and ECP.

Seventeen patients (15%) developed other liver abnormalities, including isolated hyperbilirubinemia (n=2), elevated liver enzymes (n=12) or both (n=3). No liver biopsies were performed. Presumed etiologies included conditioning-related toxicity (n=8), drug-induced liver toxicity (n=5), cytokine-release syndrome (n=3) and engraftment syndrome (n=1). All events resolved.

### Liver stiffness measurements and VOD prediction

Median liver stiffness (LS) values were 5.0 kPa (IQR 4.1–6.0) at baseline (n=104), 5.2 kPa (IQR 4.1–6.7) at day +14 (n=84), and 0.45 kPa (IQR 0.4-1.7) for the change between both time points (ΔLS).

Four of the five patients who developed VOD had normal baseline LS values. One patient with a prior diagnosis of non-alcoholic fatty liver disease and prior exposure to ponatinib and L-asparaginase had a baseline LS greater than 14 KPa compatible with advanced fibrosis (F4) (22). The median time from day +14 LS to VOD diagnosis was 13 days (range 3–21.5).

**Figure 2:**
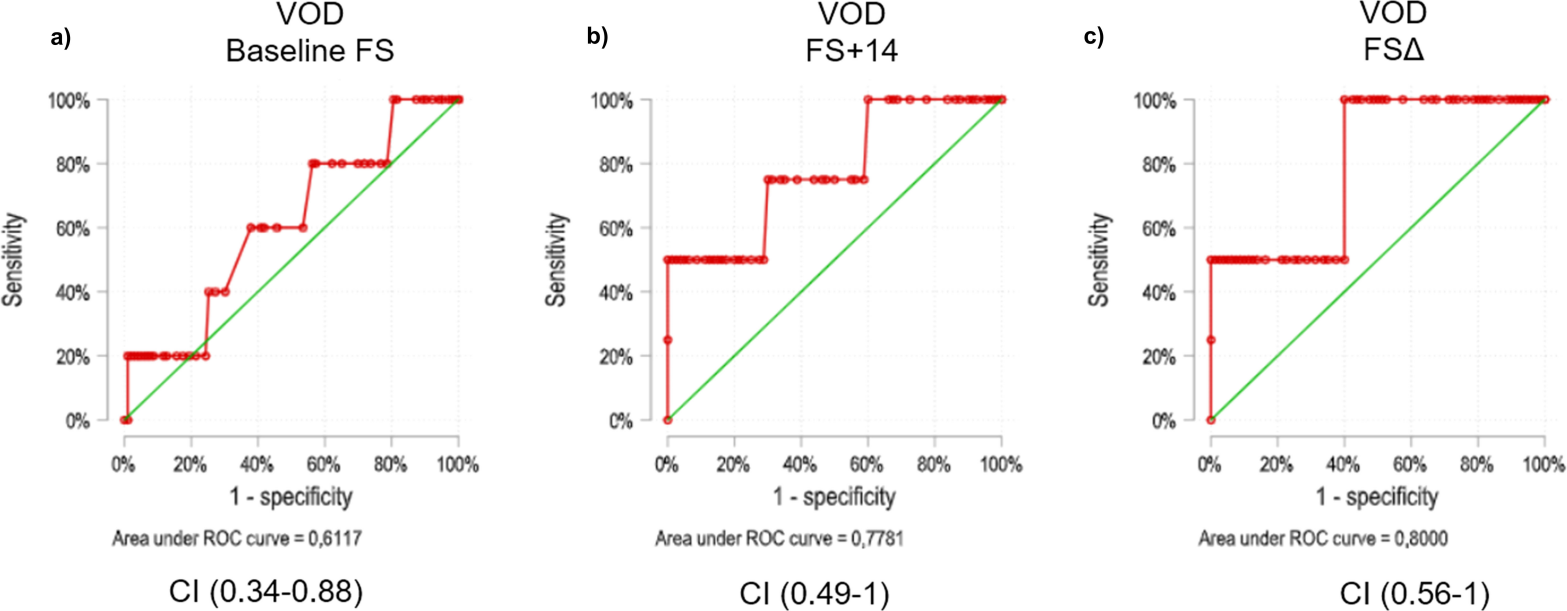
VOD prediction capability of baseline LS **(a)**, LS + 14 **(b)** and LSΔ **(c)** ROC Curves. Red line represents study observations, green line represents theoretical minimum for statistical significance. LS, liver stiffness. FS, Fibroscan; VOD, veno-occlusive disease.

**Figure 3:**
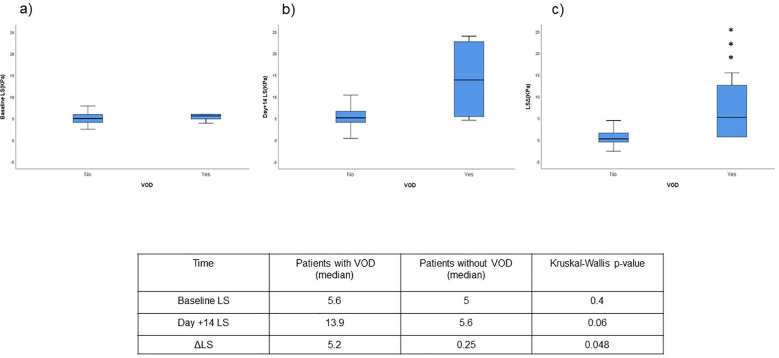
Liver stiffness variation through different timepoints. Median line of box plotsrepresents median values of LS at different timepoints, baseline **(a)**, day+14 **(b)** and LSΔ **(c)**. The top and bottom lines of the box represent interquartile range. Asterisk represent median differences reach statistical significance in Kruskal-Wallis test. LS, liver stiffness; VOD, veno-occlusive disease.

There were no significant differences in the median value of baseline LS [median 5.6 KPa (IQR 4.4-10.15) vs median 5 KPa (IQR 4.1-6); p=0.4; AUROC 0.612 (0.343-0.880)] and day +14 [median 13.9 KPa (IQR 5-23.3) vs median 5.1 KPa (IQR 4.1-6.6); p=0.06; AUROC 0.778 (0.496-1.000)] between patients developing VOD and those who did not. However, statistically significant differences were found in LSΔ between patients who developed VOD and those who did not [5.2 KPa (IQR 0.7-14.05) vs 0.25 KPa (IQR -0.5-1.65) p=0.048 (**Figure 2**); AUROC 0.8 (0.567-1)]. A LSΔ value of 6.6 KPa was chosen according to Youden Y-index as the best cut-off point for VOD prediction as it showed the best predictive performance yielding a high specificity (95%) and negative predictive value (97.4%) with a sensitivity of 50% and positive predictive value of 33.3% (**Figure 3**).

**Figure 4:**
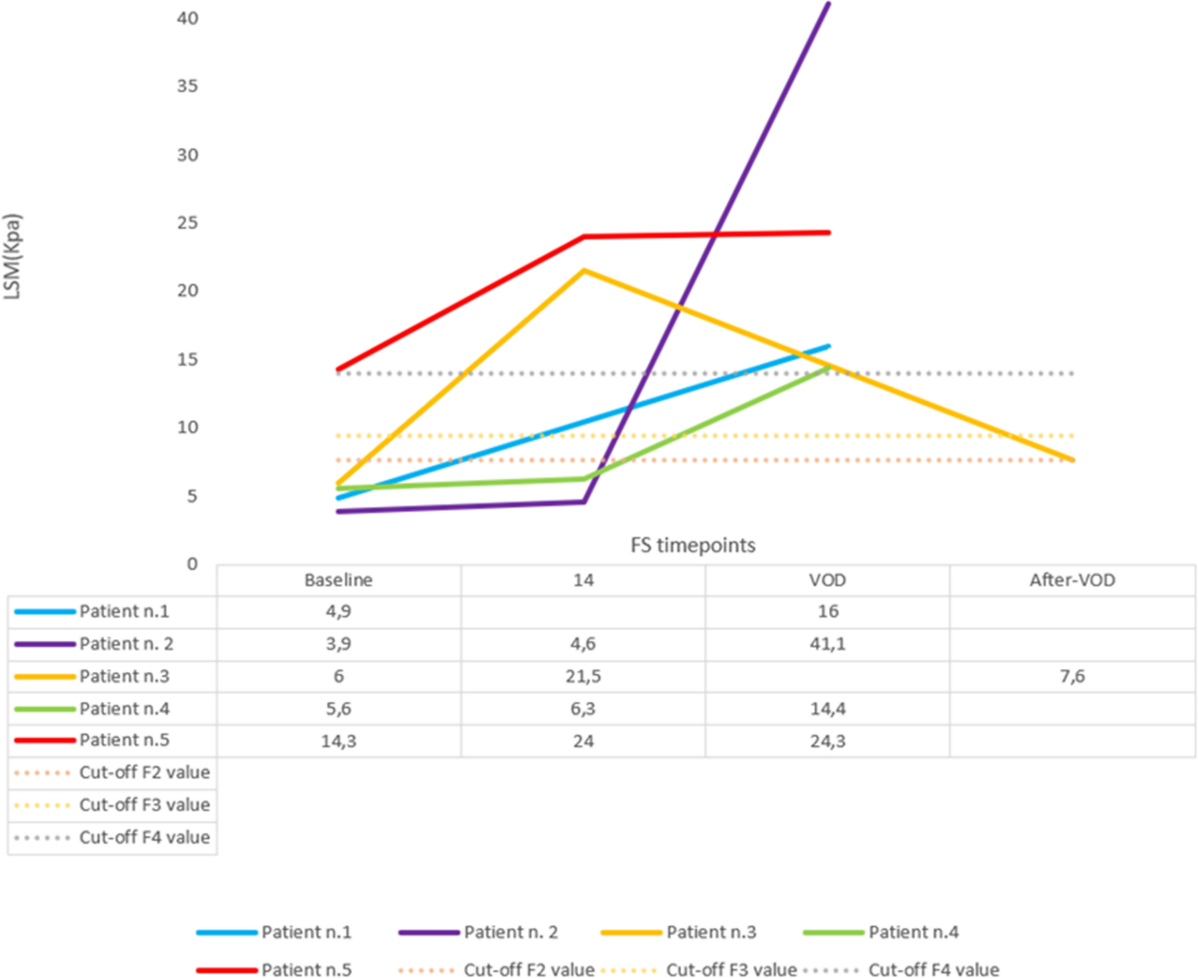
Liver stiffness measurement variation throughout different FS timepoints for patients with VOD. Y-axis accounts for LS, which is represented in Kpa. X-axis represents different timepoints for LS measurement performance: at baseline, at day+14, at the time of VOD and sometime after resolution. Solid line represents LS evolution.

In 4 out of 5 patients who developed VOD an additional TE at the time of VOD diagnosis was performed, showing a median LS of 20.15 KPa (IQR 15.6-28.5). (**Table 2**, **Figure 4****).** Only one patient, who developed VOD along with significant fluid overload resulting in moderate ascites at onset was unable to undergo the assessment.

### Liver stiffness and transplant outcomes

The predictive value of LS for OS, EFS, NRM, GRFS and relapse was assessed. The optimal cut-off established by Harrell C-index was 6.0 Kpa for both baseline and +14 LSM. The number of patients exceeding this cut-off was 26 (24%) at baseline and 28 (25.9%) at day +14.

**Figure 5:**
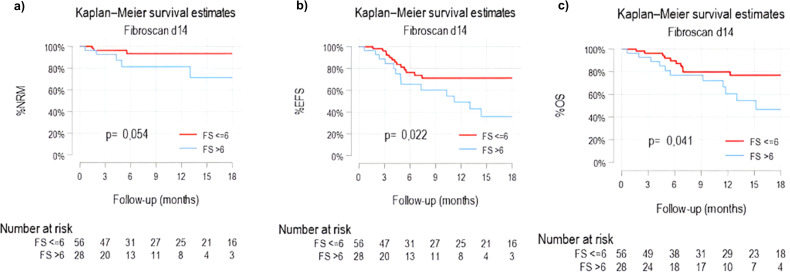
Kaplan-Meier curves for non-relapse mortality (NRM) **(a)**, event-free survival (EFS) **(b)** and overall survival (OS) **(c)**. Patients with FS + 14 higher than 6 Kpa are represented by the blue line, patients with FS + 14 equal or lower to 6 Kpa represented by the red line.

Univariate and multivariate analyses are summarized in **Tables 3**, **4**; **Figure 5**. An LS >6.0 kPa at day +14 was independently associated with worse EFS [adjusted subhazard ratio (asHR) 3.9, 95% CI 1.6–9.3], along with diabetes [asHR: 6.1 (1.3–27)]; CMV mismatch was also significant as protective factor for EFS [asHR: 0.2 (0.08–0.8)]. LS greater than 6 Kpa at day +14 also showed a trend toward worse OS (asHR: 2.3, 95% CI 0.9–6.0; p=0.08). Among patients with events accounting for EFS, NRM for patients with LS + 14>6Kpa and for those with normal LS + 14<6 were 38.5% and 15%, respectively (p=0.37).

**Table 3:** Results of univariate and multivariate analyses for event-free survival and overall survival.

Variables	Event-free survival				Overall survival			
	sHR (95%CI)	*P*-value	asHR (95%CI)	*P*-value	sHR (95%CI)	*P*-value	asHR (95%CI)	*P*-value
Baseline FS>6Kpa	0.5 (0.18-1.4)	0.2	–		0.8(0.2-2.3)	0.6	–	
FS +14>6 KPa	2.38 (1.1-5)	**0.02**	3.9 (1.6-9.3)	**0.002**	2.3 (1-5.5)	**0.045**	2.3 (0.9-6)	0.08
Prior SCT	1.37 (0.5-3.5)	0.6	–		2.7 (1-6.4)	**0.03**	2 (0.0-6.6)	0.2
Prior allogenic SCT	1.16 (0.2-7.5)	0.16	–		0.7 (0.1-5.7)	0.07	–	
Gender male	2.1 (0.8-1.9)	**0.05**	1.2 (0.4-3.6)	0.69	1.8 (0.7-4.4)	0.7	–	
RIC conditioning	1.23(0.5-2.8)	0.6	–		2.4 (0.8-7.5)	0.1	–	
Haploidentical donor	1.86 (0.9-3.9)	**0.09**	0.5-2.7	0.6	2.2 (0.9-5.4)	0.07	1.4 (0.5-4)	0.4
Age>40 years old	0.64(0.2-1.5)	0.3	–		0.5 (0.2-1.2)	0.1	–	
Psychiatric disturbance	1.6 (0.6-3.9)	0.3	–		0.8 (0.2-2.8)	0.7	–	
Cerebrovascular disease	1.5(0.3-6.1)	0.5	–		0.9 (0.1-6.2)	0.9	–	
Cardiac disease	0.8 (0.2-3.8)	0.8	–		0.5 (0.06-4.8)	0.5	–	
Lung disease	1.39 (0.6-2.8)	0.3	–		1.6(0,7-3.7)	0.2	–	
HBV positivity	0.4 (0.06-2.57)	0.3	–		NS	Ns	–	
Liver function test abnormality	0.87 (0.4-1.9)	0.7	–		1 (0.4-2.5)	0.9	–	
Diabetes mellitus	3.7 (1.2-11)	**0.015**	6.1 (1.3-27)	**0.017**	5.5 (1.9-15.7)	**0.001**	13 (3.3-51)	**0.0001**
BMI>35	0.5 (0.1-3)	0.49	–		1.6 (0.4-5.5)	0.4	–	
Rheumatologic disease	3.4 (0.5-21)	0.1	–		1.6 (0.1-17)	0.7	–	
Prior solid tumor	0.5 (0.1-1.7)	0.3	–		1.1 (0.3-3.2)	0.8	–	
Infection	1.5 (0.2-1.3)	0.3	–		1.5 (0.5-4.5)	0.4	–	
Donor >35 years old	0.6 (0.29-1.3)	0.2	–		0.7 (0.3-1.7)	0.5	–	
Female donor to male receptor	1.9 (0.9-4.1)	**0.07**	1.2 (0.3-3.6)	0.6	1.1 (0.2-1.8)	0.7	–	
CMV serostatus mismatch	0.4 (0.15-1.1)	**0.08**	0.2 (0.08-0.8)	**0.02**	0.6 (0.2-1.8)	0.4	–	
Remission	1. (0.4-2.3)	0.9	–		1(0.4-2.6)	0.9	–	
High/very high DRI	1.8 (0.9-3.7)	**0.07**	1.3 (0.6-3)	0.4	2.08 (0.9-4.6)	**0.07**	1.89 (0.8-4.4)	0.1

Bold values represent statistical significance.

**Table 4:** Results of univariate and multivariate analyses for non-relapse mortality and relapse.

	Non relapse mortality				Relapse			
Variables	sHR (95%CI)	*P*-value	asHR (95%CI)	*P*-value	sHR (95%CI)	*P*-value	asHR (95%CI)	*P*-value
Baseline FS>6Kpa	2.1 (0.6-7.4)	0.22	–		NE	–	–	
FS +14>6 KPa	3.7 (0.8-15)	**0.07**	3.3(0.7-14)	0.1	1.7(0.7-4.2)	0.2	–	
Prior SCT	1.8(0.3-8.8)	0.4	–		1.1 (0.3-3.4)	0.8	–	
Prior allogenic SCT	0.3 (0.05-2.2)	0.25	–		NE	–	–	
RIC conditioning	1 (0.2-4.1)	0.9	–		1.12(0.4-2.9)	0.8	–	
Haploidentical donor	4.1(0.89-19)	**0.069**	2.6(0.5-13)	0.2	1.4(0.6-3.4)	0.39	–	
Age>40 years old	0.39 (0.1-1.5)	0.18	–		0.68 (0.2-1.9)	0.4	–	
Psychiatric disturbance	0.7 (0.09-5.2)	0.7	–		2(0.7-5.7)	0.1	–	
Cerebrovascular disease	NS	–	–		2.22 (0.5-8.9)	0.2	–	
Cardiac disease	1.4 (0.1-13)	0.76	–		0.58 (0.1-4.1)	0.59	–	
Lung disease	1(0.3-3.5)	0.9	–		1.77 (0.75-4.2)	0.19	–	
HBV positivity	NS	–	–		0.54 (0.09-3.67)	0.56	–	
Liver function test abnormality	1.2 (0.3-4.6)	0.76	–		0.67 (0.2-1.9)	0.45	–	
Diabetes mellitus	3.2 (0.5-20)	0.2	–		3.7(0.7-17.7)	**0.09**	5.6 (1.18-26.7)	**0.03**
BMI>35	1.5 (0.2-9.9)	0.6	–		NE	–	–	
Rheumatologic disease	5.1(0.5-48.4)	0.15	–		2.44 (0.2-20)	0.41	–	
Prior solid tumor	0.5(0.07-4.5)	0.6	–		0.5 (0.1-2.1)	0.35	–	
Infection	NS	–	–		2.39 (0.9-6.2)	**0.07**	3.1 (1.1-8.8)	**0.03**
Donor >35 years old	1.2(0.3-4)	0.7	–		0.5 (0.2-1.3)	0.17	–	
Female donor to male receptor	0.9 (0.2-4.79)	0.9	–		2.42 (1-5.7)	**0.04**	2.4 (0.9-6.3)	0.06
CMV serostatus mismatch	0.7(0.1-3.4)	0.6	–		0.44 (0.1-1.3)	0.16	–	
Remission	0.54 (0.1-1.9)	0.3	–		1.62 (0.5-4.9)	0.39	–	
High/very high DRI	1.2 (0.3-4.7)	0.78	–		2.56 (1.1-5.8)	**0.02**	2 (0.8-4.8)	0.12

Bold values represent statistical significance.

Liver stiffness measurements (baseline, day +14, and ΔLS) were not predictive of acute or chronic hepatic GVHD, nor other liver-related events. These findings are illustrated in **Supplementary Figures 1**–**3**.

## Discussion

Liver injury remains a major source of toxicity and mortality in the context of allogeneic hematopoietic stem cell transplantation (allo-HSCT), with pre-existing hepatic dysfunction recognized as a significant morbidity factor prior to transplantation. In recent years, advanced modalities beyond conventional biochemical assessment have emerged for evaluating liver damage, including LS measured by TE. While these techniques are well integrated into clinical practice for other liver conditions such as viral hepatitis, their application in allo-HSCT, particularly in platforms using PTCY, is limited.

Prior studies have evaluated the role of LS measured by several techniques (FS, ShearWave and ARFI) for the prediction of allo-HSCT outcomes, mostly with conventional GVHD prophylaxis and in the context of matched donor transplant. These studies have suggested a predictive value of the increase of LSM at day +14 prior to the appearance of VOD (4–6, 9), and a potential of baseline LS for the prediction of hepatic complications and survival (7, 8, 10). A recently published study has shown the high diagnostic value of a stepwise diagnostic algorithm for VOD at the onset of clinical signs (6). In addition, LS also discriminated between VOD and other hepatic complications (6, 9).

Our study presents a real-world prospective cohort of 108 patients undergoing PTCY-based allo-HSCT including both HLA-matched and haploidentical donors, in which LS measured by TE was incorporated into a pre-specified monitoring protocol at baseline and day +14. Global incidence of hepatic events was low, with an incidence of VOD of less than 5% despite the use of an additional alkylating agent, and also a low incidence of acute and chronic hepatic GVHD of 4.6 and 7.6% was observed. In our study, the increase of LS higher than 6.6 KPa from baseline to day+14 seemed to increase likelihood of upcoming VOD, and LSM greater than 6 KPa at day +14 was predictive for event-free survival, underscoring the potential relevance of LS dynamics for predicting transplant outcomes in the context of PTCY-based allo-HSCT.

Prior studies demonstrating the potential of LSM for VOD prediction and its utility in differentiating VOD from other post-transplant hepatic toxicities were based on intensive monitoring schedules involving multiple FS assessments per patient, an approach that is challenging to implement in routine clinical settings. In contrast, our study utilized a simplified protocol involving two scheduled FS assessments: at baseline and on day +14 post-transplant. This approach shows liver stiffness increases prior to VOD becomes clinically evident and may anticipate the development of VOD, with a median time to event of 13 days, comparable to prior reports (5). Because the assessment was scheduled for day +14 and the median onset of VOD occurred later, the development of ascites did not limit the feasibility of performing the examination in most cases. Low incidence of VOD precluded from proving correlation between LSΔ and VOD severity, which could have added more strength to our approach. In this cohort, the relatively late median onset of VOD (day +27), possibly related to PTCY use, may have reduced the predictive sensitivity of the day +14 LSM.

Our study demonstrate consistent LS performance in patients uniformly receiving PTCY-based GVHD prophylaxis, a key distinction from earlier research, which included limited numbers of patients on PTCY and predominantly those receiving conventional GVHD prophylaxis (4 – 7, 9). Additionally, this study included a higher proportion of haploidentical donor transplants, in contrast to the mostly HLA-matched donor populations of previous cohorts (8). Despite the presence of established risk factors for VOD (namely the use of high-dose alkylating agents and donor mismatch), the incidence of VOD in this cohort was low (5%), aligning with trends observed in other recent studies. This likely reflects increased awareness and the implementation of safer transplant strategies (23, 24). Similarly, the incidence of GVHD was low, likely due to the widespread use of PTCY, which may have limited the statistical power to assess FS predictive value for this complication.

Hepatic comorbidity, as defined by the hematopoietic cell transplantation-specific comorbidity index (HCT-CI), has been recently recognized as a major determinant of non-relapse mortality (NRM) in the PTCY setting, while other comorbidities appear to have limited prognostic relevance (15). In our study, a LS + 14 >6 kPa was associated with inferior OS and EFS in univariate analysis and remained significantly associated with poorer EFS in multivariate analysis. Although supported by prior studies, selecting day +14 for LS assessment is somewhat arbitrary (6–10). Whether other timepoints could better reflect hepatic function dynamics or enhance prognostic value, as seen with evolving biomarkers like EASIX, remains unknown (23, 24).

This cohort showed a relatively low incidence of NRM, reflecting continued improvements in HSCT outcomes. These favorable rates, however, may have limited the ability to detect associations between FS measurements and NRM. Additional limitations include the small number of hepatic and GVHD events, that may have introduced risk for spurious associations considering the number of primary endpoints. Limitations also include incomplete second FS measurements in some patients because of both technical and clinical reasons potentially introducing bias, and potentially confounded multivariate findings, such as the association between diabetes and poorer outcomes, likely influenced by age and DRI. In addition, there is a lack of external validation for the LS cut-off described. Finally, regarding the LS value on day +14 in patients who developed VOD, some of the measurements obtained were extreme and in one case the data could not be obtained. Therefore, although the value of the test for VOD prediction is promising, our findings need to be confirmed in larger patient cohorts. Despite these constraints, this study constitutes the largest prospective evaluation of TE in a uniformly treated PTCY-based allo-HSCT population. It suggests the prognostic value of FS for VOD and LS + 14 >6 kPa as a potential early marker of poorer EFS. Further studies are required to provide external validation and confirm these results.

In conclusion, LSM via FS demonstrated predictive value for clinical outcomes in patients undergoing PTCY-based allo-HSCT. LS + 14 represents a dynamic, non-invasive, and reproducible measure that could enhance current HSCT risk stratification strategies. Further studies are warranted to identify the optimal timing for FS assessments and to clarify its role in predicting specific hepatic complications.

## Data Availability

The raw data supporting the conclusions of this article will be made available by the authors, without undue reservation.

## References

[1] Snowden JA Sánchez-Ortega I Corbacioglu S Basak GW Chabannon C de la Camara R . Indications for haematopoietic cell transplantation for haematological diseases, solid tumours and immune disorders: current practice in Europe, 2022. Bone Marrow Transplant. (2022). doi: 10.1038/s41409-022-01691-w, PMID: 35589997 PMC9119216

[2] Castéra L Vergniol J Foucher J Le Bail B Chanteloup E Haaser M . Prospective comparison of transient elastography, Fibrotest, APRI, and liver biopsy for the assessment of fibrosis in chronic hepatitis C. Gastroenterology. (2005) 128:343–50. doi: 10.1053/j.gastro.2004.11.018, PMID: 15685546

[3] Berzigotti A . Non-invasive evaluation of portal hypertension using ultrasound elastography. J Hepatol. (2017) 67:399–411. doi: 10.1016/j.jhep.2017.02.003, PMID: 28223101

[4] Colecchia A Marasco G Ravaioli F Kleinschmidt K Masetti R Prete A . Usefulness of liver stiffness measurement in predicting hepatic veno-occlusive disease development in patients who undergo HSCT. Bone Marrow Transplant. (2017) 52:494–7. doi: 10.1038/bmt.2016.320, PMID: 27941774

[5] Colecchia A Ravaioli F Sessa M Alemanni VL Dajti E Marasco G . Liver stiffness measurement allows early diagnosis of veno-occlusive disease/sinusoidal obstruction syndrome in adult patients who undergo hematopoietic stem cell transplantation: results from a monocentric prospective study. Biol Blood Marrow Transplant. (2019) 25:995–1003. doi: 10.1016/j.bbmt.2019.01.019, PMID: 30660772

[6] Ravaioli F Colecchia A Peccatori J Pagliara D Grassi A Barbato F . Diagnostic accuracy of liver stiffness measurement for the diagnosis of veno-occlusive disease/sinusoidal obstruction syndrome after hematopoietic stem cell transplantation (HSCT), the ELASTOVOD STUDY: an investigator-initiated, prospective, multicentre diagnostic clinical trial. Bone Marrow Transplant. (2025) 60:978–93. doi: 10.1038/s41409-025-02570-w, PMID: 40253530

[7] Auberger J Graziadei I Clausen J Vogel W Nachbaur D . Non-invasive transient elastography for the prediction of liver toxicity following hematopoietic SCT. Bone Marrow Transplant. (2013) 48:159–60. doi: 10.1038/bmt.2012.113, PMID: 22705804

[8] Karlas T Weber J Nehring C Kronenberger R Tenckhoff H Mössner J . Value of liver elastography and abdominal ultrasound for detection of complications of allogeneic hemopoietic SCT. Bone Marrow Transplant. (2014) 49:806–11. doi: 10.1038/bmt.2014.61, PMID: 24710567

[9] Debureaux P-E Bourrier P Rautou P-E Zagdanski A-M Boutiny M Pagliuca S . Elastography improves accuracy of early hepato-biliary complications diagnosis after allogeneic stem cell transplantation. haematol. (2020) 106:2374–83. doi: 10.3324/haematol.2019.245407, PMID: 32732366 PMC8409044

[10] Karlas T Weiße T Petroff D Beer S Döhring C Gnatzy F . Predicting hepatic complications of allogeneic hematopoietic stem cell transplantation using liver stiffness measurement. Bone Marrow Transplant. (2019) 54:1738–46. doi: 10.1038/s41409-019-0464-x, PMID: 30809042

[11] Davidov Y Shem-Tov N Yerushalmi R Hod T Ben-Ari Z Nagler A . Liver stiffness measurements predict Sinusoidal Obstructive Syndrome after hematopoietic stem cell transplantation. Bone Marrow Transplant. (2024) 59:1070–5. doi: 10.1038/s41409-024-02288-1, PMID: 38658660 PMC11296942

[12] Sorror ML Storb RF Sandmaier BM Maziarz RT Pulsipher MA Maris MB . Comorbidity-age index: A clinical measure of biologic age before allogeneic hematopoietic cell transplantation. J Clin Oncol. (2014) 32:3249–56. doi: 10.1200/JCO.2013.53.8157, PMID: 25154831 PMC4178523

[13] D’Souza A Fretham C Lee SJ Arora M Brunner J Chhabra S . Current use of and trends in hematopoietic cell transplantation in the United States. Biol Blood Marrow Transplant. (2020) 26:e177–82. doi: 10.1016/j.bbmt.2020.04.013, PMID: 32438042 PMC7404814

[14] Bailén R Pascual-Cascón MJ Guerreiro M López-Corral L Chinea A Bermúdez A . Post-transplantation cyclophosphamide after HLA identical compared to haploidentical donor transplant in acute myeloid leukemia: A study on behalf of GETH-TC. Transplant Cell Ther. (2022) 28:204. doi: 10.1016/j.jtct.2022.01.020, PMID: 35108627

[15] Bolaños-Meade J Hamadani M Wu J Al Malki MM Martens MJ Runaas L . Post-transplantation cyclophosphamide-based graft-versus-host disease prophylaxis. N Engl J Med. (2023) 388:2338–48. doi: 10.1056/NEJMoa2215943, PMID: 37342922 PMC10575613

[16] Spyridonidis A Labopin M Savani BP Kulagin A Blaise D Broers AEC . The impact of individual comorbidities in transplant recipients receiving post-transplant cyclophosphamide. Bone Marrow Transplant. (2025) 60:499–506. doi: 10.1038/s41409-025-02514-4, PMID: 39910175

[17] Armand P Kim HT Logan BR Wang Z Alyea EP Kalaycio ME . Validation and refinement of the disease risk index for allogeneic stem cell transplantation: a study from the CIBMTR. Blood. (2014), blood–2014-01-552984. doi: 10.1182/blood-2014-01-552984, PMID: 24744269 PMC4047501

[18] Harris AC Young R Devine S Hogan WJ Ayuk F Bunworasate U . International, multicenter standardization of acute graft-versus-host disease clinical data collection: A report from the mount sinai acute GVHD international consortium. Biol Blood Marrow Transplant. (2016) 22:4–10. doi: 10.1016/j.bbmt.2015.09.001, PMID: 26386318 PMC4706482

[19] Jagasia MH Greinix HT Arora M Williams KM Wolff D Cowen EW . National institutes of health consensus development project on criteria for clinical trials in chronic graft-versus-host disease: I. The 2014 diagnosis and staging working group report. Biol Blood Marrow Transplant. (2015) 21:389–401.e1. doi: 10.1016/j.bbmt.2014.12.001, PMID: 25529383 PMC4329079

[20] Mohty M Malard F Abecassis M Aerts E Alaskar AS Aljurf M . Revised diagnosis and severity criteria for sinusoidal obstruction syndrome/veno-occlusive disease in adult patients: a new classification from the European Society for Blood and Marrow Transplantation. Bone Marrow Transplant. (2016) 51:906–12. doi: 10.1038/bmt.2016.130, PMID: 27183098 PMC4935979

[21] Health UD of, Services H . Common terminology criteria for adverse events (CTCAE) (2017). Available online at: https://cir.nii.ac.jp/crid/1370017279879487627 (Accessed October 6, 2024).

[22] Berzigotti A Tsochatzis E Boursier J Castera L Cazzagon N Friedrich-Rust M . EASL Clinical Practice Guidelines on non-invasive tests for evaluation of liver disease severity and prognosis – 2021 update. J Hepatol. (2021) 75:659–89. doi: 10.1016/j.jhep.2021.05.025, PMID: 34166721

[23] Gómez-Centurión I Morillo AIG Martínez AP Calvo MC Chinea A González L . Sinusoidal obstruction syndrome/veno-occlusive disease after unmanipulated haploidentical hematopoietic stem cell transplantation with post-transplantation cyclophosphamide: A study on behalf of the spanish hematopoietic stem cell transplantation and cellular therapy group (GETH). Transplant Cell Therapy Off Publ Am Soc Transplant Cell Ther. (2024) 30:914.e1–8. doi: 10.1016/j.jtct.2024.06.003, PMID: 38851323

[24] Ruutu T Peczynski C Houhou M Polge E Mohty M Kröger N . Current incidence, severity, and management of veno-occlusive disease/sinusoidal obstruction syndrome in adult allogeneic HSCT recipients: an EBMT Transplant Complications Working Party study. Bone Marrow Transplant. (2023) 58:1209–14. doi: 10.1038/s41409-023-02077-2, PMID: 37573397 PMC10622315

