# Supplementary material for: Prognostic value of liver stiffness measurement for complications after allogeneic transplant with post-transplant cyclophosphamide
Source: Front Immunol. 2026 Jan 23;16:1699219. doi: 10.3389/fimmu.2025.1699219 (PMC12875988; doi:10.3389/fimmu.2025.1699219)
Supplement: Supplementary file 1 [file Table1.docx]

**SUPPLEMENTARY MATERIAL**

**Supplementary table 1. Components of the univariate analysis.**

| Patients (n=108) | |
| --- | --- |
| Demographic of donors and patients (n, %)   - Donor sex mismatch (female donor to-male receptor) - Donor age more than 35 years old - Patient age>40 years old - Patient gender male | 10 (9.2)  49 (45.6)  90 (83.3)  58 (53.7) |
| Transplant characteristics (n, %)   - Prior allo-HSCT - Prior SCT - Haploidentical donor/mismatched unrelated - High or very high Disease risk index - Reduced intensity of conditioning - CMV mismatch - Active disease at HSCT | 13 (12)  8 (7.4)  55 (50.9)  80 (74)  32 (29.7)  24 (22.2)  80 (74.1) |
| Comorbidites included in HCT-CI (n, %)   - Psychiatric disturbance - Cerebrovascular disease - Cardiac disease - Lung disease - HBV positivity - Prior solid tumor - Active infection - Liver function test abnormality - Diabetes mellitus - Body mass Index>35 - Rheumatologic disease | 15 (13.8)  4 (3.7)  6 (5.5)  46 (42.6)  7 (6.5)  15 (13.8)  28 (25.9)  5 (4.6)  9 (8.3)  3 (2.7) |
| Elastographic variables (n, %)   - Basal LSM>6 KPa - Day+14 LSM>6 KPa | 26 (24.1)  28 (35.9) |

Abbreviations: CMV, Cytomegalovirus; HSCT, hematopoietic stem cell transplant; HCT-CI, Hematopoietic Cell Transplantation-Comorbidity;

**
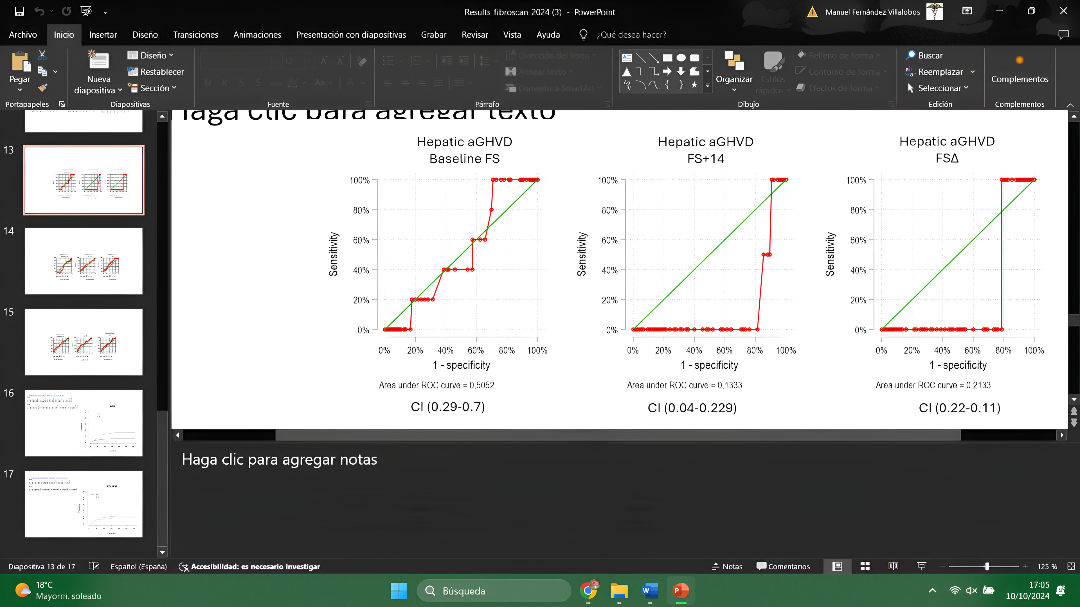
**

**Supplementary figure 1. Hepatic acute GVHD prediction capability of baseline FS(A), FS +14(B) and FSΔ(C). ROC Curves** line represents study observations, green line represents theoretical minimum for statistical significance. FS, Fibroscan; aGVHD, acute graft-versus-hostage disease.

**
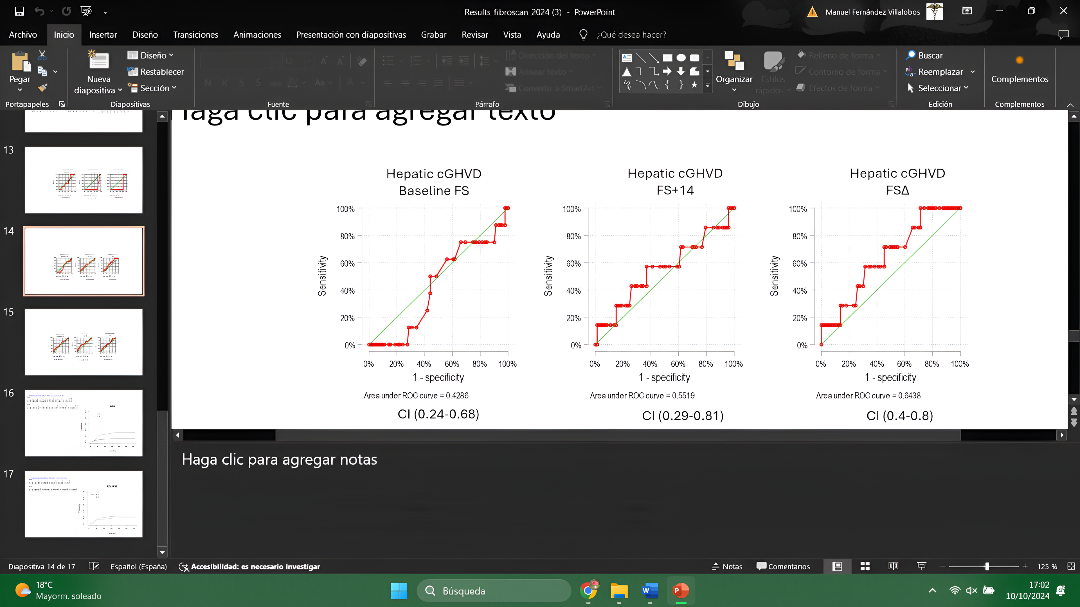
Supplementary figure 32 Hepatic chronic GVHD prediction capability of baseline FS(A), FS +14(B) and FSΔ(C). ROC Curves.** Red line represents study observations, green line represents theoretical minimum for statistical significance. FS, Fibroscan; cGVHD, chronic graft-versus-hostage disease.

**
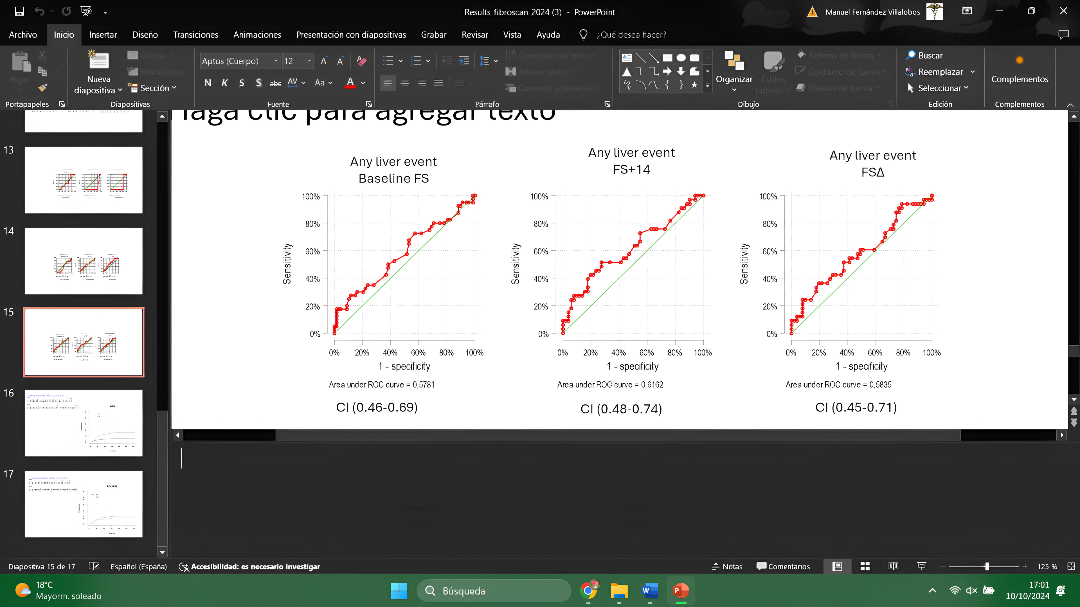
**

**Supplementary figure 3. Liver event prediction capability of baseline FS(A), FS +14(B) and FSΔ(C). ROC Curves** Red line represents study observations, green line represents theoretical minimum for statistical significance. FS, Fibroscan.
